# Intergenerational differences in social support for the community‐living elderly in Beijing, China

**DOI:** 10.1002/hsr2.96

**Published:** 2018-10-17

**Authors:** Yang Cheng, Jing Xi, Mark W. Rosenberg, Siyao Gao

**Affiliations:** ^1^ Faculty of Geographical Science Beijing Normal University Beijing P. R. China; ^2^ Department of Geography and Planning, Queen's University, K7L 3N6 Ontario Canada

**Keywords:** Beijing, community‐living elderly, intergenerational difference, qualitative study, social support

## Abstract

**Background and Aims:**

The combination of the rapid process of social‐economic development, urbanization, and population ageing brings many challenges for care providers and quality of life of the community‐living elderly in Beijing, China. This research aims to understand the intergenerational differences of social support for the elderly in the socio‐cultural context of Beijing.

**Methods and Results:**

To answer this research question, we collected 30 semi‐structured in‐depth interviews from elders aged 60 and over in three communities in Beijing. The constant comparative method was used for analysis. The results show that the young‐old (people aged 60 to 74) received more formal social support and less informal social support compared to their parents' generation. The formal social support they received was not much different but they received less informal social support compared to the older‐old (people aged 75 and over) living in the same communities. The young‐old expect to receive more formal social support when they become the older‐old, as the informal social support from their children would be reduced due to the one‐child policy and socio‐cultural changes.

**Conclusions:**

Intergenerational differences of social support for the elderly do exist in the form of instrumental, financial, and emotional support. The findings help us understand how socio‐economic development and urbanization processes affect the daily life and social support of the community‐living elderly from different age groups, and also provides knowledge for improving the quality of life for the elderly in Beijing.

## 1 INTRODUCTION

The combination of the rapid process of urbanization, population ageing, and social‐economic development brings many challenges for providing social services for the elderly in Beijing, the capital of China. Limited studies have been conducted on whether these changes can have an influence on the caregiving for the elderly in China compared to the process that developed countries have experienced.^1^ The experiences of urbanization and population ageing in China are also happening in some other developing countries, which makes the challenges faced by China as a bellwether for developing countries in south Asia and Sub‐Africa regions.

The older population, aged 60 and over, increased from 1.7 million in 2000 to 2.97 million in 2014 in Beijing.^2^ The size of the elderly population is expected to increase, to 4.15 million by 2025.^3^ After the economic reform in China in 1978, the GDP per capita in Beijing was only 1,671 RMB in 1982 and increased to 115,000 RMB in 2016.^4^ Around the same period as the economic reform, by the end of 1970s, the Chinese government set up the one‐child policy (a population planning policy of China, to limit the great majority of family units to have only one child each) to control the rapid growth of population, a policy which was in place until recently. After the implementation of this policy for more than three decades, it successfully decreased the fertility rate and controlled the population growth. It, however, increased the rate of population ageing and increased the challenges for providing elderly care by family members. With the rapid socio‐economic change, traditional large‐size households gradually changed to nuclear families, as the living distance between the adult children and their parents and the proportion of female participation in full‐time jobs are increasing. The average number of family members decreased from 3.75 in 1982 to 2.56 in 2015.^5^, ^6^ The first generation of the “only child” policy is now middle‐aged and their parents are now over 60 years old. The older parents' care needs will increase in the next few decades, while the family care resources are decreasing.

As a result of these socio‐economic changes, the young‐old generation (population aged 60 to 74) faces a different situation for ageing compared to the older‐old (population aged 75 and over), and the parents' generation of the young‐old. Meanwhile, the community care and residential care services are still under development. All of these changes bring challenges for providing social support for the elderly to meet their care needs.

The Chinese government aims to improve the reduced family care resources by increasing more social care resources. The Beijing municipal government has made a series of policies to improve the availability and accessibility of social and health care services for the elderly living in communities. Meanwhile, pension system and health care system have been under reform in China in recent decades. Urban and rural residents receive different pension and health care benefits according to their household registration and status of employment. The Chinese government has established a universal non‐contributory pension plan covering urban non‐employed workers and rural residents, combined with an old‐age insurance program covering urban employees.^7^ However, the benefit amount of national social pension plan is very low, with a country average of 81 RMB (13 US dollars) per month in 2014. By the end of 2013, 95% of the population was covered by one of the medical insurance schemes. There is a significant gap of social welfare benefits between rural and urban residents.^8^

This research aims to understand the intergenerational differences in social support between the young‐old (the elderly people aged 60 to 74) and the older‐old (the elderly people aged 75 and over) in the socio‐cultural context of Beijing. Thirty elderly, including the young‐old and the older‐old people living in three communities built in 1950s, 1990s, and 2000s, were interviewed to understand this issue, which contributes knowledge aimed at increasing social support resources and improving quality of live for the elderly in Beijing. The second section describes the conceptual framework that applies in this research. The intergenerational differences of life experiences in the context of the socio‐economic changes in the past 30 years in China are explained in the third section. The methods and results are presented in the fourth and fifth sections. Conclusions and discussions are drawn in the last section of this paper.

## 2 UNDERSTANDING SOCIAL SUPPORT

Social support has been studied across a wide range of disciplines including psychology, medicine, sociology, and public health, among others.^9^ Social support has been defined as the assistance and protection given to others, especially to individuals.^10^, ^11^ In recent studies, researchers have conceptualized support in terms of the structural components and the functional components. Based on the actual support received, it can also be divided into perceived support and received support.^12^, ^13^

Social support is a multidimensional construct which can be derived from various sources, such as families, friends, neighbors, colleagues, organizations, and governments.^14^ It can be tangible in the form of financial support, assistance, and material goods, or intangible as the form of emotional support such as being listened to, understood, and comforted.^9^, ^14^, ^15^ Langford *et al*.^9^ reviewed 85 articles and summarized social support as emotional, instrumental, informational, and appraisal support. Emotional support involves the provision of caring, empathy, love, and trust.^16^, ^17^, ^18^ Instrumental support refers to the provision of financial assistance, material goods, or services.^17^, ^19^ Information support is defined as information provided by others during a time of stress, to solve problems.^17^, ^18^, ^20^ Appraisal support refers to the communication of information relevant to self‐evaluation instead of problem‐solving.^17^

Many studies have analyzed the relationship between social support and health focusing on the elderly population. Previous research suggested that having adequate social support was associated with better self‐rated health among the elderly people in the context of both the developed and developing countries.^15^, ^21^, ^22^, ^23^ With regard to mental health, Wang, Li, and Chen^24^ studied 266 empty‐nested elderly in Ningbo, China, and found that social support and mental health status of empty‐nested elderly are significantly lower than those in other elderly people. Increasing the social support of empty‐nested elderly people is important for improving their mental health.

**Figure 1:**
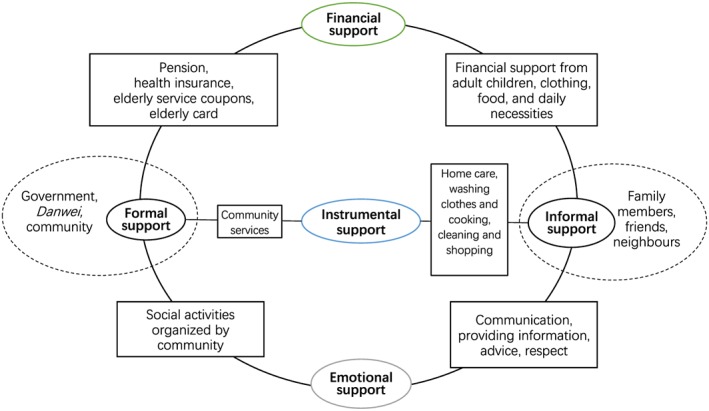
Conceptual framework of social support

The social support that elderly people receive is influenced by important events in their life‐span, such as retirement and loss of one's spouse.^25^ In addition, the quantity and quality of social support, which is called social embeddedness by Langford *et al.,* 9 can also affect elderly people's life satisfaction and subjective well‐being.^15^, ^26^, ^27^, ^28^, ^29^ However, little research has been done to understand the intergenerational difference in social support in the context of rapid urbanization and socio‐cultural changes in China. Modified conceptual framework is needed to adapt for this case study in Beijing. In this study, we propose the following framework, which categorizes social support as formal support and informal support depending on the source of social support. The former refers to the support from governments, enterprises, organizations, and communities, while the later refers to the support from families, neighbors, and friends. In terms of attributes, social support is categorized as instrumental support, financial support, and emotional support. Instrumental support refers to caregiving and other support to manage one's daily life, such as shopping, cleaning, laundry, and cooking activities. Financial support refers to the financial and material support. Emotional support refers to mental support, such as listening, talking, consulting, and caring (Figure 1).

## 3 INTERGENERATIONAL DIFFERENCES IN LIFE EXPERIENCES

As the socio‐economic environment has changed rapidly in China since the socio‐economic reform in the late 1970s, the intergenerational differences have dramatically increased. People's perceptions on filial piety are also changing. The elderly's expectation on receiving care from their children is decreasing, while the young generation's willingness to provide care for their parents is still strong.^30^ However, many of the younger generation provide financial support and emotional support to their older parents to compensate the limited instrumental care that they can provide.^30^

In the young‐old's generation, the urban youth during the Cultural Revolution (1966–1976) were sent to live and work in agrarian areas to be re‐educated by the peasantry, which is referred to as “up to the mountains and down to the countryside movement”. During the movement, many urban youths at that time lost their opportunity for higher education.^31^ When they returned to the cities after the Cultural Revolution, the economic reform from central planning to market economy started. Meanwhile, the one‐child policy was introduced in 1979 to encourage the young generation to marry at later ages and urban families to have only one child. This policy was formally phased out in 2015.^32^ From the mid‐1990s to 2004, the Chinese central government restructured the state‐owned enterprises (SOEs) sector to increase the efficiency of industry and be more competitive in the global economy. The middle‐aged and older workers (40–60 age groups), those with low educational levels, and females were more likely to be laid‐off during the reform.^33^

The state‐owned sector before the reform provided medical care, pensions, housing, and child education and continuing job training.^28^ After the reform, the *danwei* system (*danwei* refers to a place of employment in the context of state‐owned sector during the planned economy of China) gradually collapsed. The laid‐off workers lost their employment and some of the welfare they used to have, which resulted in many of them living under the poverty line.^34^ Together with the economic reform, China has also gone through a reform in social welfare systems, including a health care and pension reform as well as the commercialization of housing in urban areas. With the rapid economic development in China, the young generation has been able to take advantages of market opportunities through education and training, while the laid‐off workers were facing economic hardship.

Many of the laid‐off are entering their old age and their disadvantageous position in the socio‐economy challenges the social support they can receive. Together with the rapid urbanization process, the living distance between the older parents and the adult children increases, which also has an impact on the family support they can receive, especially among the parents of the one‐child generation. Therefore, with limited educational opportunity, one‐child policy, SOEs reform, social welfare reform, commercialization of housing, collapse of *danwei* system, and rapid urbanization process overlap, which is the case for the young‐old generation, those who are at disadvantageous positions are less likely to receive social support.

With regard to the older‐old, they were born before the foundation of People's Republic of China and suffered during the World War II. They survived the “Three Years of Natural Disasters”, between the years of 1959 and 1961, characterized by widespread famine national‐wide.^35^ The majority of them were either not employed or retired before the SOEs reform. The socio‐economic reform has improved people's quality of life, but at the same time, the older‐old are not the population who enjoy the most benefits from the reform. Their adult children play an important role in providing various forms of social support for them.

## 4 DATA AND METHODS

**Figure 2:**
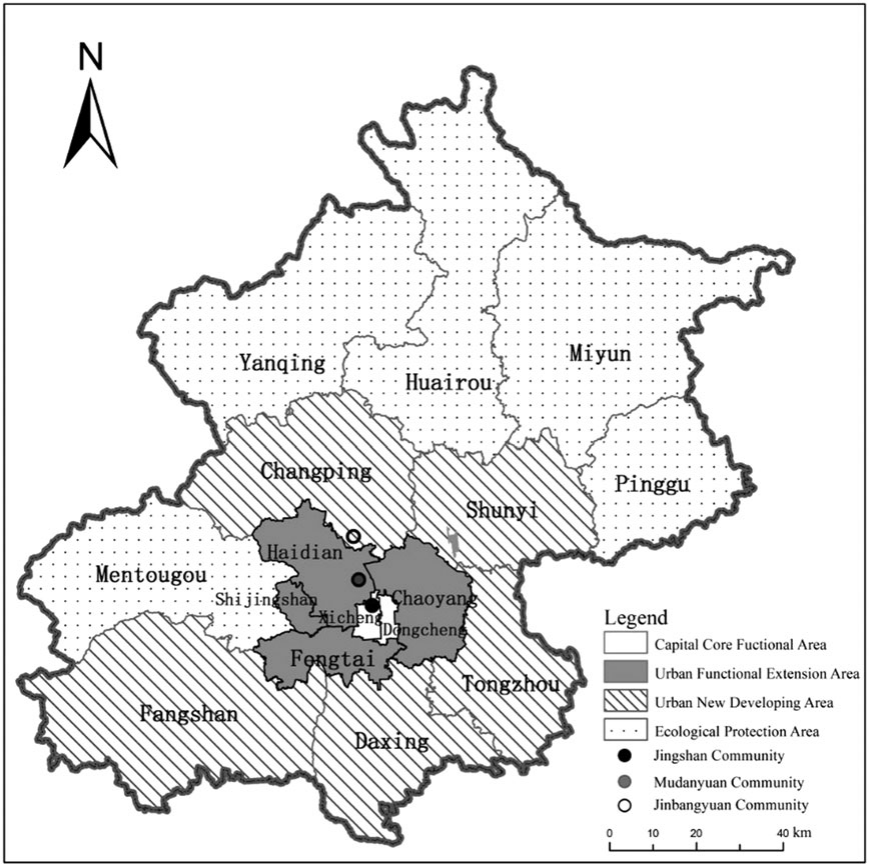
Map of Beijing, showing the locations of the three case study sites

This study collected 30 semi‐structured in‐depth interviews from the elderly aged 60 and over in three communities in Beijing. The three studied communities are typical in Beijing in terms of their locations and living environments. Jingshan Community (A) is a traditional community located in the Core Functional Area of Beijing. There are 2,958 residents with Beijing household registration, living in 128 court‐yards in this community. There are 780 people (26.37% of the total population registered in this community) aged 60 and over and 149 people aged 80 and over. Mudanyuanxili Community (B) was built in the 1990s and is located between the third and the fourth ring roads in the Urban Function Extension Area of Beijing. There are 1,669 residents with Beijing household registration, living in four six‐floor and six 21‐floor apartment buildings in this community. There are 462 people (27.68% of the total population registered in this community) aged 60 and over and 84 people aged 80 and over. Jinbangyuan Community (C) was built in the 2000s and is located in the Urban New Development Area of Beijing. This is the largest community among the three study sites, with 3,906 residents who have Beijing household registration, living in 28 six‐floor apartment buildings. There are 732 people (18.74% of the total population registered in this community) aged 60 and over and 56 people aged 80 and over. The income level of residents in Community B and C are higher than Community A. Community A and B are located in the urban area of Beijing, while Community C is located in the suburban area of Beijing. The locations of the three case study communities are shown in Figure 2.

The interviews were conducted between April and September in 2015. Officials of the subdistrict (a form of township‐level division which is part of a larger urban area) were first contacted by visiting. After permission was given by the subdistrict's administrative agency, interviewers went to the open space of the communities, where older people like to gather together for chatting or excising, to recruit participants. Only the elderly with Beijing household registration, and those who were willing and capable to communicate were enrolled in this study. Purposive selecting in the community considering the age and sex, and snowball sampling by recommendation from the participants were used for recruiting participants. Five young‐old aged 60 to 74 and five older‐old aged 75 and over were interviewed in each community, with 30 participants in total in the three communities (Table 1). As the older people were cautious about conducting interviews at their private homes, they were interviewed in public spaces such as the benches in the green space or parks in the communities with which they were familiar. Interviewers started the interviews based on the question list after obtaining oral informed consent from the interviewees. The young‐old were asked about their care experiences for their older parents, the social support their parents received, their own care needs and social support received, and the perceptions on their future care and social support. The older‐old were asked about their current situation of health status, care needs, and social support they received. The interviewer took notes, and the interviews were audio recorded at the same time. Mandarin was the language used during the interviews because it is the official and everyday language in Beijing. The total time for interviewing was 904 minutes, with around 30 minutes for each participant. Ethics approval for the research project was obtained from Beijing Normal University in China. All participants in the present study gave their informed consent before their inclusion in the study. Details that might disclose the identity of the subjects under study were omitted and all data collected were de‐identified.

**Table 1:** Information on the participants

No.	Age	Sex	Educational level	Occupation	Living arrangement	No. of children
A01	64	M	Junior school	Worker	With spouse	1
A02	65	F	Junior school	Bus conductor	With spouse	1
A03	69	M	College	Cadre	With spouse and son	2
A04	70	M	High school	Cadre	With spouse	1
A05	70	F	Junior school	Worker	With spouse	1
A06	77	F	Uneducated	Housewife	With spouse	3
A07	78	M	Uneducated	Worker	With spouse	3
A08	79	F	Technical secondary school	Librarian	With daughter	3
A09	85	F	Primary school	Hairdresser	Living alone	1
A10	86	F	Uneducated	Accountant	With spouse	4
B01	60	F	High school	Accountant	With spouse	1
B02	66	F	High school	Cadre	With spouse and son	2
B03	70	F	Uneducated	Peasant	With son	3
B04	70	F	University	Engineer	With spouse	1
B05	72	M	High school	Cadre	With spouse	1
B06	75	F	Uneducated	N/A	Living alone	4
B07	80	F	Uneducated	Housewife	Living alone	5
B08	82	M	University	Senior lecturer	With spouse	2
B09	86	F	Junior school	Worker	Living alone	4
B10	91	M	Primary school	Worker	With spouse and daughter	4
C01	65	F	Junior school	Accountant	With spouse	1
C02	68	M	Technical secondary school	Cadre	With spouse	1
C03	73	F	Primary school	Office worker	With spouse	3
C04	74	M	Junior school	Cadre	With spouse	2
C05	74	F	Junior school	Worker	With spouse	1
C06	77	F	Primary school	Accountant	With son	1
C07	78	F	Medical school	Anesthetist	With spouse	1
C08	82	M	Uneducated	Soldier	With spouse and son	6
C09	82	F	Uneducated	Housewife	Living alone	4
C10	90	M	Primary school	Postman	With spouse and daughter	4

Analysis of the data is based on the constant comparative method.^36^ The content of the audio recordings was fully transcribed. The transcripts were read line‐by‐line several times in order to mark the key points with a series of codes, and similar codes were grouped into a concept. As concepts accumulated and their descriptions became more detailed, similar concepts were rearranged by common themes, and thematic categories were developed. Finally, the thematic categories were used to define concepts and find associations between themes. The thematic categories, concepts, and resulting associations were used to provide explanations for the findings.^37^, ^38^

## 5 RESULTS

Life course is important for understanding the social support that the elderly receive. Participants talked about the important events during the life course of their generation, which they think significantly affect their current socio‐economic status. The parents of the young‐old participants in this study had all passed away. The caregiving they offered to their parents had taken place between 1980s and 2000s.

*We are the most unlucky generation. When we were at the age of studying, the Cultural Revolution started, then we had to go to the countryside to work. After we came back to the cities, the one‐child policy was implemented, and we were laid‐off due to the economic reform. Our generation, the ones born in the 1950s, was the unluckiest people as we suffered all these challenges*. (B‐01, 60 years old)

It was common for the young‐old's generation to view themselves as the unlucky generation. After the collapse of the old *Danwei* work unit system at the end of 1990s, the new *Community* system has been under reconstruction to pull together resources from various sectors to provide formal support for the older residents. The availability and accessibility to formal support varies among the three communities due to the lack of regulations and standards for community services. The participants expressed their expectations of the government to play a crucial role in monitoring the quality of services, including housekeeping work and food services for the elderly. The following parts explain the intergenerational differences from the perspectives of instrumental support, financial support, and emotional support in detail.

### 5.1 Instrumental support

The formal instrumental support includes practical support and health care services provided by communities, such as food service, housekeeping service, and home care services. The young‐old reported that there was a lack of formal instrumental support in the community they live. They mainly do the housekeeping work by themselves, as most of them are independent at this stage. The informal instrumental support from family members was reduced compared to their parent's generation and the older‐old, as many of the young‐old had only one child. For those who do not live with their adult children, the children come to visit them once a week at the most, and some children visit their parents only once every two or three months. Instead of receiving informal instrumental support from their adult children, the young‐old who live together with their adult children provide instrumental support for their adult children's family, such as doing housekeeping work, preparing meals, and taking care of the grandchildren.

*I have six siblings and my parents lived with me. Me and my wife provided quite a lot of instrumental support for my parents and my siblings took turns to come to help every week or two weeks … Now I live with my wife. My son (the only one child of the interviewee) and his family live in Chaoyang (another district in Beijing). They come to visit once a week. We don't need their instrumental support at the moment, but they cannot offer much even when we need such support in the future … More support should be provided by the government and community, especially for people like us with only one child and those whose children live far away*. (A‐01, 64 years old)

When the young‐old were asked about their future perceptions on instrumental support, they talked about the impact of the one‐child policy and the challenges they face. They do not have much expectation for increasing informal instrumental support, as they do not want to increase the care burden on their only child who is already overloaded by childcare and their daily work tasks. The young‐old expect more formal instrumental support when they become the older‐old and their demands for instrumental support increase.

*You have your living style, and the children have theirs. I try to manage my own business, and do not make troubles for my children. Good health is better than anything else*.(A‐06, 77 years old)

They also talked about the challenges in hiring nannies. Some of them had negative experiences when hiring nannies, while some of them are not comfortable with caregiving offered by strangers. The young‐old expects the government to improve the food services, housekeeping services, and health care services at the community level.

The instrumental support for the parents' generation of the young‐old was mainly informal support from themselves. Little instrumental support was provided by the *Danwei* work units or communities from the 1980s to the 2000s. The young‐old recalled how they got balance between their work and caring for their parents. In many cases, their parents were living with one of the adult children's family, and other siblings shared the responsibilities of providing instrumental care by frequent visits. For those who lived alone, the family members took turns to provide instrumental support.

The older‐old also reported they tried to do the housekeeping work by themselves for as long as possible. They had to depend on their adult children or hire temporary workers by the hour when their self‐care ability declined. Their adult children provided informal instrumental support for them in the form of grocery shopping and house cleaning, by frequent visits, or relocating the older parents into their own households. Some of the older‐old's children had retired and they take the responsibility of caring for their older parents. The older‐old usually has more than one child; the adult children take turns to offer informal instrumental support which decreases the care burden loaded on each adult child.

*I have lived in this community since 1995. My son's family also lives in this community … In recent years, my daughter cooks and delivers the meals for me. She lives quite close to here … She and my granddaughter hire temporary workers by the hour to clean my apartment … My son's family also offers support*. (B‐06,75 years old)

### 5.2 Financial support

Both the young‐old and the older‐old reported that formal financial support increased due to the pension reform. The major income source of the retired young‐old is their pension, which can generally cover their living expenses. The pension provided them with the capability to be less depended on informal financial support from their adult children. However, their income would not be enough if there was any additional cost such as inpatient health care expenses. The cost would be expensive, even though 85% of the expenses can be reimbursed from their health care insurance. They were also worried about the high cost of hiring a nanny when they need instrumental support. With an average income, they would have difficulties in affording a full‐time nanny.

*My parents did not have pension and their income source was mainly from us (their adult children) … I gave my parents part of my salary every month … My income source is from my pension. I have 3300 RMB and my husband has 4000 RMB every month. My daughter does not give us money, but she always buys us groceries … I have several chronic diseases and my health care insurance cannot cover all the cost. It can be quite expensive sometimes … I am worried about the future, when I lose my self‐care ability and the health care expenses will increase … I won't be able to afford moving into a residential care facility or hiring a nanny*. (A‐02, 65 years old)

*My income is mainly from my pension which is around 2000 RMB per month. My children buy me food and clothes, but they do not give me cash. I am enrolled in the “One old one young” health insurance and 70 per cent of my hospitalization expenses can be reimbursed. I use my savings to pay for the rest. My children put money together if my savings cannot cover (the health care expenses)*. (A‐10, 86 years old)

For those who were not employed before, they can receive 300 to 800 RMB per month from the uniform pension, and they were covered by Urban Resident Basic Medical Insurance. However, they were likely to have financial difficulties when their partners passed away. As a result, they moved from financial independence to be dependent on their adult children.

*I only receive 300 RMB from the basic living allowance every month and I mainly depend on my sons and daughter. My daughter buys me clothes and groceries … My health insurance does not cover much on the outpatient cost. My elder son pays for it … My husband used to have pension which was around 4000 RMB. We did not financially depend on the children. Now is different after my husband passed away*. (C‐08, 82 years old)

With regard to the parents' generation of the young‐old, little formal financial support was received by the young‐old's parents if they were not employed before. Most of their financial support came from the informal support by their children. The young‐old mentioned that the health care expenses of their parents used to be a heavy burden for the family, as there was not a universal health insurance system that can cover everyone in their parents' generation. The family members had to share the cost of health care.

*The income source of my parents was from me and my siblings. There was no pension insurance and health insurance. We paid for my parents' food and clothes. My salary was only 30 RMB in the 1970s and I gave 3 to 5 RMB to my parents. I earned more in the 1980s and 1990s, then I gave 30 to 40 RMB to my parents*. (A‐01, 64 years old)

The young‐old also reported that the formal social support their parents received was tied to their former workplace. Employees worked in SOEs, and public institutions received different welfare in terms of pension and health care in their parents' generation. There were great differences between the elderly who were unemployed, workers in factories, and employees in public institutions.

*My father was teaching in a college … When he was diagnosed with cancer (in the 1980s), his college sent a staff to care for him during the daytime … My mother was working in the hospital. After the diagnose of my father, the hospital provided him with a single room. My mom was able to care for him without quitting her job. All the health care expenses were reimbursed. There was no financial burden for us at that time*. (C‐01, 65 years old)

The young‐old expect their financial security to be improved as they become the older‐old. They expect the increase in formal financial support will be able to cover all of their expenses and the cost of their increasing demands for instrumental support and health care services. As the parents of the one‐child generation, they do not have much expectation on the increase in informal financial support from their children. The older‐old reported that they receive informal financial support from their adult children in the form of money, clothing, food, and daily necessities.

*My wife and I both have pensions. We are quite healthy and don't go to see the doctor often... We have only one child and don't want to put too much pressure on him … It would be great if the community care services are improved. I hope the services include food service, housekeeping service, and home care … The residential care facilities with good service are quite expensive, over 5000 RMB per month. We cannot afford to it … We hope the formal support increases to help us access these services*. (C‐02, 68 years old)

### 5.3 Emotional support

The formal emotional support for the young‐old comes from various social activities, such as gatherings, lectures, outgoing, singing, and dancing groups organized by the communities and their former workplace. The young‐old reported that the formal emotional support increased compared to their parents' generation, as the community increasingly organizes social activities. The informal emotional support from their children and neighbours, however, decreased compared to their parents' generation and the older‐old living in the same community.

*The neighbours were all Beijing local residents in the past. We understood each other and cared about each other. The neighbours were like families. If my neighbor's relatives came and there was no place to stay, they just came to stay in my home. Now is different. People become selfish, especially these migrants. The society has changed a lot*. (A‐05, 70 years old)

Comparing to the older‐old, the young‐old receive less informal emotional support from their children. Instead, former colleagues and friends are significant providers for their informal emotional support. The older‐old always depends on their adult children for taking them to meet with their former colleagues and friends due to the decrease in their mobility. As a result, the older‐old prefer communicating with their neighbors within the communities, and the young‐old prefer doing some exercises, dancing, and going out with their friends.

*My children are busy. They come to visit once or twice a month. I often contact my former colleagues. We chat on the phone and we get together sometimes. Not as often as before. I spend most of my time at home and in the community as my health status declines*. (C‐04, 74 years old)

*I don't go out of the community now. I don't know the way to get back if I go out. My mental health has declined especially after my husband passed away. I just come out of my apartment, sit down on the bench and chat with the neighbors in the garden. Sometimes I watch other older people dance and exercise in the open space. I cannot join in such activity now*. (C‐08, 82 years old)

The young‐old expect more formal emotional support to maintain or improve their mental well‐being when they become the older‐old. Being different from the informal instrumental support and financial support, the young‐old expect more informal emotional support from their children in their older age. In their mind, the reducing informal instrumental support and financial support can be compensated by the increasing formal support. However, the informal emotional support from the adult children cannot be substituted by the formal emotional support.

*We don't have many social activities in the community. It will be good if they organize some activities for the older residents in the community … I don't want to put pressure on my daughter. I am just happy that she often comes to visit me and chats with me. You know, this is important for the older people*. (A‐02, 65 years old)

The older‐old also talked about the instrumental support they used to provide for their grandchildren, but little support was provided to them by the grandchildren when they grew up. They expect emotional support from their grandchildren, and they were disappointed by the lack of emotional support from their grandchildren.

*My grandchildren are grown up. I used to look after them when they were young. Now they are busy and seldom come to visit. Even when they come, they just stay for a while and have a meal here*. (A‐09, 85 years old)

## 6 DISCUSSION AND CONCLUSION

To summarize, our results show that the young‐old receive more formal social support and less informal social support compared to their parents' generation. Comparing to the older‐old living in the same communities, the formal social support the young‐old received is not much different, even considering their age difference, but they receive less informal social support than the older‐old. Part of the reason is that the demands for informal support, especially instrumental support, are less among the young‐old than the older‐old. The findings show that the socio‐cultural changes including family size, living distance between older people and their children's family, and view of filial piety affect both the adult children and the young‐old's behavior of giving and receiving social support. The young‐old expect to receive more formal social support when they become the older‐old, to compensate the reduced informal social support from their children. The majority of the young‐old expect for developments in community care services in the future.

The social welfare system, including pension system, minimum living allowance, and health care insurance provides a safety net for the elderly. The formal support has, in general, improved. Many of the young‐old's parents heavily depended on their adult children for living expenses.

Informal emotional support from former colleagues was important for the elderly. Gathering, group tour, and phone calls were the ways through which they maintain their friendships. Some participants reported that they had a stronger sense of belonging to the former workplace than the community, and kept a strong social network with their former colleagues even though they did not live in the same community. However, as their mobility decreases, the elderly become more dependent on their families for emotional support.

The social welfare policy, family planning policy, and urban relocation compensation policies all affect the social support that the elderly receive. The survey in 2010 showed that 93.7% of the urban older residents received pensions.^39^ Pensions enable the elderly to gain more financial independence and discourse in decision making. The improvements in providing community services including food service, housekeeping service, and social activities are important for providing formal instrumental and emotional support for the elderly.

Social changes have great impacts on the life course of the elderly by affecting their educational level, family structure, household size, and economic status, which further influence the social support that the elderly receive. The experiences of going to countryside losing educational opportunities, having only one child in each family, being laid‐off from the state‐owned enterprises, being relocated due to urbanization, and undergoing the social reform of pension and health care systems have dramatic impacts on the socio‐economic status, family structures, and social welfare of the young‐old's generation. All of these impacts will bring more challenges for the elderly in one or two decades, when the health status of the young‐old's generation declines and they need more social support for elderly care. Many old buildings were tore down for commercial use or building expensive apartment buildings in the central city during the urbanization process. The elderly whose residence has been demolished were relocated to a new living environment. The change of the living environment also affects the social support they receive in both positive and negative ways, such as improvement in the physical living environment and lose in familiarity and sense of belonging to the old community.

The rapid socio‐economic change in China also affects the change of cultural norms on filial piety and elderly care, which has an impact on social support. For the young‐old, they have a strong sense of filial piety and think of providing various forms of social support to the elderly as their obligation. They overcame challenges by frequently visiting their parents and providing instrumental and emotional support for their parents. Along with the rapid socio‐economic change, the absolute authority of the older parents decreases, and the meaning of filial piety also changes. The young generation is willing to provide social support to the parents but in the way of financial support to compensate for their unavailability in providing instrumental support, as they lack the time or they live far away from their parents. The elderly also understand the challenge for their adult children to provide care for them as they did for their parents. They have less such expectation of their adult children and expressed their worries about their future, which was also found by Luo, Shi and Xiao.^30^

Household income directly affects the elderly's financial support, and the high housing price in Beijing has also indirectly affect their social support. The income level influences whether the elderly can afford formal instrumental support or hire caregivers. The high housing price in Beijing limited the elderly's choices on housing; many of them can only live in the traditional communities, with a relatively poor living environment, and their adult children live at a relatively long distance from them, which reduces the availability of their adult children to provide instrumental care for them. This is the new challenge that the young‐old face which their parents did not experience.

Previous literature in English‐speaking countries have reported similar findings to those reported in this study. First, older people with disadvantaged socio‐economic status experience reduced social support.^40^, ^41^ Secondly, geographical separation from family and friends may increase the risk of isolation, depression, and loneliness, and reduce the social support that older people receive.^42^, ^43^, ^44^ Thirdly, the change of living environment such as gentrification challenges the social support older people receive. Gentrification may result in forced physical relocation, increased housing costs, and homeownership exclusion. These changes threaten neighborhood social spaces, sense of belonging, and social cohesion.^44^, ^45^ There are also similarities to the situation in other developing countries. A study in Thailand^46^ found that the migration of young adults to urban areas creates a geographic mismatch in the locations of older individuals and their children, which affect the social support the older people receive. Emotional and financial support can be provided remotely through modern communication technology. However, meal preparation, personal care, and transportation require regular and frequent services, and, thus, closer proximity makes the services easier. In the future, children may no longer be able to be the main support and caregivers for parents due to demographic changes.^46^ There is, however, limited research on the intergenerational difference of social support that older people receive. The findings of this research contribute understanding of the change in social support in terms of instrumental, financial, and emotional support from the formal and informal sources in the context of developing country.

There are some limitations of this study. All of the participants in this study are older‐people with Beijing household registration. Older migrants are increasing in Beijing due to various reasons such as caring for grandchildren or relocating closer to their family members. They are ineligible to receive some social welfare and social services due to the household registration. This study is unable to provide information on social support received by the floating older population in Beijing. In future studies, this group of older population should be considered.

## FUNDING

This work was supported by the National Natural Science Foundation of China (NO. 41301164 and NO.41671497).

## CONFLICTS OF INTEREST

None declared.

## AUTHOR CONTRIBUTIONS

Conceptualization: Yang Cheng, Mark Rosenberg

Investigation: Yang Cheng, Jing Xi

Methodology: Yang Cheng, Jing Xi

Supervision: Yang Cheng, Mark Rosenberg

Writing‐Original Draft Preparation: Yang Cheng, Jing Xi, Siyao Gao

Writing‐Review & Editing: Yang Cheng, Mark Rosenberg, Siyao Gao

## References

[1] Knickman JR , Snell EK . The 2030 problem: caring for aging baby boomers. Health Serv Res. 2002;37(4):849‐884. 12236388 10.1034/j.1600-0560.2002.56.x PMC1464018

[2] Committee on Ageing of Beijing . (2015) Report on Elderly Population Information and Development of Elder Care of Beijing in 2014. Beijing, China. Available at: http://zhengwu.beijing.gov.cn/tjxx/tjgb/t1412150.htm (accessed on 26/7/2017). (In Chinese).

[3] Beijing Municipal Bureau of Statistics . The Future 50 Years of Beijing Municipality. Beijing: China: Electronics Press; 2000 (In Chinese).

[4] Beijing Municipal Bureau of Statistics . (2017) Available at: http://www.bjstats.gov.cn/tjsj/tjgb/ndgb/201702/t20170227_369468.html (accessed on 1/8/2017) (In Chinese).

[5] Beijing Municipal Bureau of Statistics . Beijing Statistical Yearbook 1983. Beijing, China: China statistical press; 1983 (In Chinese).

[6] Beijing Municipal Bureau of Statistics . Beijing Statistical Yearbook 2016. Beijing: China: China Statistical Press; 2016 (In Chinese).

[7] Liu T , Sun L . Pension reform in China. J Aging Soc Policy. 2016;28(1):15‐28. 26549002 10.1080/08959420.2016.1111725

[8] Cheng Y , Yu J , Rosenberg MW . Rural‐urban China and the changing older population, in Routledge In: Dhirathiti N, Higo M, Klassen T, eds. Handbook on Ageing and Old‐Age in Asia‐Pacific. London: Routledge; 2018:46‐63.

[9] Langford C , Bowsher J , Maloney J , Lillis P . Social support: a conceptual analysis. J Adv Nurs. 1997;25(1):95‐100. 9004016 10.1046/j.1365-2648.1997.1997025095.x

[10] Shumaker SA , Bronwell A . Toward a theory of social support: closing conceptual gaps. J Soc Issues. 1984;40(4):11‐33.

[11] Wortman CB , Dunkel‐Schetter C . Conceptual and methodological issues in the study of social support In: Baum A, Singer JE, eds. Handbook of Psychology and Health, Lawrence Erlbaum Associates. NJ: Hillsdale; 1987:63‐108.

[12] Gaveras EM , Kristiansen M , Worth A , Irshad T , Sheikh A . Social support for south Asian Muslim parents with life‐limiting illness living in Scotland: a multiperspective qualitative study. BMJ Open. 2014;4(2):e004252. 10.1136/bmjopen-2013-004252 PMC3918973 24503303

[13] Reblin M , Uchino BN . Social and emotional support and its implication for health. Curr Opin Psychiatry. 2008;21(2):201‐205. 18332671 10.1097/YCO.0b013e3282f3ad89 PMC2729718

[14] Lin C , Li L , Ji G , Jie W . Emotional social support and access to care among elderly living with HIV in rural China. Int J Geriatr Psychiatry. 2015;30(10):1041‐1047. 25663571 10.1002/gps.4260 PMC4527961

[15] Dai Y , Zhang C , Zhang B , Li Z . Social support and the self‐rated health of older people: a comparative study in Tainan Taiwan and Fuzhou Fujian province. Medicine. 2016;95(24):e3881. 27310979 10.1097/MD.0000000000003881 PMC4998465

[16] Cronenwett LR . Network structure, social support, and psychological outcomes of pregnancy. Nurs Res. 1985;34(2):93‐99. 3844737

[17] House JS . Work Stress and Social Support. Reading, MA: Addison‐Wesley; 1981.

[18] Krause N . Understanding the stress process: linking social support with locus of control beliefs. J Gerontol. 1987;42(6):589‐593. 3680876 10.1093/geronj/42.6.589

[19] Tilden VP , Weinert SC . Social support and the chronically ill individual. Nurs Clin North Am. 1987;22(3):613‐620. 3649795

[20] Cutrona CE , Russell DW . Type of social support and specific stress: Toward a theory of optimal matching In: Sarason BR, Sarason IG, Pierce GR, eds. Social Support: An interactional view. New York: John Wiley and Sons; 1990:319‐366.

[21] Auslander G , Litwin G . Social networks, social support, and self‐ratings of health among the elderly. J Aging Health. 1991;3(4):493‐510.

[22] Chen H , Meng T . Bonding, bridging, and linking social capital and self‐rated health among Chinese adults: use of the anchoring vignettes technique. PLoS One. 2015;10(11):e0142300 10.1371/journal.pone.0142300 26569107 PMC4646615

[23] Hurtado D , Kawachi I , Sudarsky J . Social capital and self‐rated health in Colombia: the good, the bad and the ugly. Soc Sci Med. 2011;72(4):584‐590. 21185633 10.1016/j.socscimed.2010.11.023

[24] Wang Y , Li A , Chen Z . Correlational study on the social support and mental health of empty nester older adults. J Daqing Normal University. 2007;6:35‐41. (In Chinese).

[25] Uchino B . Understanding the links between social support and physical health: a life‐span perspective with emphasis on the separability of perceived and received support. Perspect Psychol Sci. 2009;4(3):236‐255. 26158961 10.1111/j.1745-6924.2009.01122.x

[26] He Z . Socioeconomic status and social support network of the rural elderly and their physical and mental health. Soc Sci China. 2002;3:l35. (In Chinese).

[27] Li J . Social support and quality of life of the elderly in China. Popul Res. 2007;3(13):50‐60. (In Chinese).

[28] Wu F , Huang N . New urban poverty in China: economic restructuring and transformation of welfare provision. Asia Pacific Viewpoint. 2007;48(2):168‐185.

[29] Zheng Z , Chen G . The impact of social support on the elderly's sense of happiness. J Jinan University. 2005;15(5):83‐85. (In Chinese).

[30] Luo Y , Shi W , Xiao Y . Change trends, existing challenges and countermeasures of the modes of elderly care for aged urban residents. J Xi'an Jiaotong University (Social Sciences). 2013;33:78‐84. (In Chinese).

[31] Pan MX . An assessment of the ‘up to the mountains, down to the village’ movement. Sociological Stud. 2005;05:154‐181. (In Chinese).

[32] Xinhua Net (2015) *Communique of the Fifth Plenary Session of the 18th Communist Party of China (CPC) Central Committee* Available at: http://news.xinhuanet.com/politics/2015-10/29/c_1116983078.htm (accessed on 1/8/2017). (In Chinese).

[33] He S , Wu F , Webster C , Liu Y . Poverty concentration and determinants in China's urban low‐income neighbourhoods and social groups. Int J Urban Reg Res. 2010;34(2):328‐349. 20726146 10.1111/j.1468-2427.2010.00907.x

[34] Gomersall K , Wang M . Life‐course experiences of laid‐off workers and their attitudes towards migrant workers in urban China. Int Dev Plan Rev. 2013;35(3):219‐240.

[35] Xu H , Wei JF . Demographic Bonus, population age structure and Chinese ageing population. Ecol Econ. 2014;30(3):16‐20. (In Chinese).

[36] Glaser BG , Strauss AL . The discovery of grounded theory. Chicago, US: Aldine; 1967.

[37] Strauss A , Corbin J . Basics of qualitative research: Grounded theory procedures and techniques. Newbury Park, US: Sage; 1990.

[38] Strauss A , Corbin J . Basic of qualitative research: Techniques and procedures for developing Grounded Theory. Thousand Oaks, US: Sage; 1998.

[39] Du P . An analysis on the health status of the older persons in China. Popul Econ. 2013;6:3‐9. (In Chinese).

[40] Melchiorre MG , Chiatti C , Lamura G , Torres‐Gonzales F , Stankunas M , et al. Social support, socio‐economic status, health and abuse among older people in seven European countries. PLoS One. 2013;8(1). e54856 10.1371/journal.pone.0054856 PMC3559777 23382989

[41] Weyers S , Dragano N , Mobus S , Beck EM , Stang A , et al. Low socioeconomic position is associated with poor social networks and social support: results from the Heinz Nixdorf recall study. Int J Equity Health. 2008;7(1):13‐19. 18457583 10.1186/1475-9276-7-13 PMC2424055

[42] Burns VF , Lavoie JP , Rose D . Revisiting the role of neighbourhood change in social exclusion and inclusion of older people. J Aging Res. 2012;2012:1‐12. 10.1155/2012/148287 PMC3195441 22013528

[43] Finlay JM , Kobayashi LC . Social isolation and loneliness in later life: a parallel convergent mixed‐methods case study of older adults and their residential contexts in the Minneapolis metropolitan area, USA. Soc Sci Med. 2018;208:25‐33. 29758475 10.1016/j.socscimed.2018.05.010

[44] Versey HS . A tale of two harlems: gentrification, social capital, and implications for aging in place. Soc Sci Med. 2018;214:1‐11. 10.1016/j.socscimed.2018.07.024 30125754

[45] Smith R , Lehning A , Kim K . Aging in place in gentrifying neighborhoods: implications for physical and mental health. Gerontologist. 2018;58(1):26‐35. 28958016 10.1093/geront/gnx105

[46] Rittirong J , Prasartkul P , Rindfuss RR . From whom do older persons prefer support? The case of rural Thailand. J Aging Stud. 2014;31:171‐181. 25456634 10.1016/j.jaging.2014.10.002

